# Informed Consent in Otolaryngologic Surgery: Case Scenario from a Nigerian Specialist Hospital

**DOI:** 10.1155/2014/120985

**Published:** 2014-07-08

**Authors:** O. A. Afolabi, J. O. Fadare, O. T. Ajiboye

**Affiliations:** ^1^Department of Ear, Nose, and Throat, University of Ilorin, PMB 1515, Ilorin 240213, Nigeria; ^2^University of Ilorin Teaching Hospital, PMB 1459, Ilorin 240212, Nigeria; ^3^Department of Pharmacology, Ekiti State University, PMB 5363, Ado-Ekiti 360221, Nigeria; ^4^Accident and Emergency Department, University of Ilorin Teaching Hospital, PMB 1459, Ilorin 240212, Nigeria

## Abstract

Informed consent is a foundational concept necessary for ethical conduct of clinical research and practice. It is a technical tool that shifts the autonomy to decide whether a medical procedure should be performed—from the doctor to the patient. However there is an ongoing discussion in bioethical circles on the level of comprehension of the informed consent process by the patients and research participants. We present this case vignette and the discussion afterwards to explore the question of to what extent a patient comprehends the information given to him/her before a surgical procedure is carried out. In other words, the question being asked here is how informed is informed consent in the context of oto-laryngological practice.

## 1. Background

The acquisition of medical information before undergoing any surgical operation is a complex process. It is increasingly being recognized that the patient plays an active role rather than just passively accepting doctors' advice [1, 2]. Studies on this subject have largely yielded nonsatisfactory results [3]. Informed consent itself is a technical tool that shifts the autonomy to decide whether a medical procedure or clinical research should be performed—from the doctor to the patient [4]. Increase in the awareness of different websites has increased nonmedical sourcing of information being utilized by patients [5]. In this era of increased patient expectations, the medical profession is trying to optimize information delivery by using patient decision software [6] and printed information sheets (in up to 25% of ENT departments) [7] and increasingly by shifting the role of provision of patient information to more senior members of the team [8, 9]. Some studies in the United States have argued that the federal government poses an actual barrier to informed consent concerning oral contraceptives and induced abortion [10]. The understanding of informed consent among patients in developing countries like Nigeria depends on the educational level, extended family culture, rural-urban dichotomy, and religion [11]. The purpose of the study was to contribute to the debate on the level of comprehension and information retention during the process of informed consent using a case vignette from otolaryngological surgical practice. To our knowledge, this is the first report on informed consent report from north-central Nigeria.

## 2. Case Scenario

Patient AA is a 26-year-old female undergraduate who was seen in ENT clinic in a Nigerian specialist hospital with complaints of nasal discharge, nasal obstruction, excessive sneezing, mouth-breathing, and occasional cough.

Physical examination revealed a young lady who was not pale, anicteric, and afebrile. Examination of the nasal cavity revealed normal shaped nose and no skin changes. There was an obvious pale, multiple polypoid bluish mass partially obliterating both nasal cavities which did not bleed on contact. The mass arises from the lateral wall; both turbinates appear bluish and engorged with partial patency. Neck examination revealed discrete submandibular and submental lymph nodes which were nontender.

An assessment of chronic rhinosinusitis with bilateral allergic nasal polyp was made on clinical ground. Patient had X-rays of the paranasal sinuses which revealed opacity of both maxillary sinuses and nasal cavities and this supported the above clinical findings. Patient could not afford computerized tomography due to financial challenges. The computerized tomographic scan is an important radiologic investigation that would have provided information on the possibility of offering the patient endoscopic sinus surgery along with ethmoidectomy and sphenoidotomy and possibly surgery to the frontonasal recess as the anatomy of the ethmoid and its immediate relations will be known. Other baseline investigations were done and found to be normal.

The details of what surgery is going to be done was explained to the patient in the clinic as well as the anaesthesia, the recovery process, postoperative management, and complication that can arise from the surgery. She was admitted and a written informed consent was signed by the patient and witnessed by a ward nurse after another round of discussion of the surgery about to be performed.

Patient had endoscopic guided bilateral nasal polypectomy and intranasal antrostomy under general anaesthesia after counseling in the clinic and a well-documented informed and understood consent. Ethmoidectomy, middle meatal antrostomy, and frontal recess surgery could not be done as there was no computerized tomographic scan that would assist in the anatomical guidance of this region. Patient was managed with antibiotics, decongestant, analgesics, and steroid spray postoperatively. Postoperatively before discharge patient expressed satisfaction at the outcome of surgery as both nasal cavities were clear and patent.

Six months after surgery, patient returned to the clinic with recurrence of her former symptoms. She then claimed that she was not well-informed about the possibility of recurrence and the chances for a total cure of the condition. Patient then went through another period of counseling and information giving after which she still refused to have repeat surgery.

## 3. Discussion

The doctrine of informed consent reminds us to respect persons by fully and accurately providing information relevant to exercising their decision-making rights and experts include at least the following elements in their discussions of the concept [12]: provision of information: patients should have explanations, in understandable language, of the nature of the ailment or condition, the nature of proposed diagnostic steps and/or treatment(s) and the probability of their success, the existence and nature of the risks involved, and the existence, potential benefits, and risks of recommended alternative treatments (including the choice of no treatment). However there is need for assessment of the patient's understanding of the above information, the capacity of the patient or surrogate to make the necessary decision(s), and assurance that the patient has the freedom to choose among the medical alternatives without coercion or manipulation [12]. In the case scenario above the assessment of the understanding of the informed consent was not done. The goals of this consent process include the development of the patient's comprehensive understanding of the clinical situation, and the timely exercise by the patient, of active choices regarding the circumstances [13, 14]. In developing countries like Nigeria, however, most of this information is not followed to letter. Rather, most patients do not play an active role in making the choices for their healthcare, a departure from what is mostly obtained in the developed world [1, 2].

The case scenario is a case of chronic rhinosinusitis which is an inflammation of the nasal passages and contiguous sinuses complicated with polyposis. By far, the most common cause of polyps is allergy as approximately 30% of patients with nasal polyps test positive for environmental allergies, followed by chronic sinus infection [15, 16]. The patient in the case study is a 26-year-old female above the age of dependency in the developed world. However review of the limited relevant empirical data suggests that adolescents, especially those aged 18 yr and older, may have as well developed decisional skills as adults for making informed health care decisions and consent taking [17, 18]. The patient in this case study was adequately informed in the language best understood which is her local language of all the earlier mentioned components of the informed consent, and this was also done twice for the patient as she had the information in the clinic and on the ward while on admission. Previous studies have shown that preoperative information and preparedness with surgery may correlate with a successful surgical outcome [19].

Also, inadequate investigation of the patient was also contributory to the exposure of the poor understanding of the consent of the surgery done for the patient. With availability of computerized tomographic scan, patients would have had the opportunity of other treatment options which include middle meatal antrostomy, ethmoidectomy, frontal recess surgery, and sphenoidotomy.

Other studies have shown that in the developed world most medical information is usually sourced for from nonmedical sources like the internet to consolidate what they have been told by the physician or get additional information that was not explained by the physician [5–9, 16]. This however is not the case in the developing world, Nigeria included, as these sources of information are not widely available there. Also, there is a widespread traditional belief in our society that “the physician knows best” and patients therefore accept all information without any queries; this is likely to have affected the patient being discussed. Most patients only want the lesion to be removed and once this is done and they are symptom-free, many of them do not bother to see the physician except when there is a recurrence. This, we believe, is as a result of the disease process on the part of the patients and inadequacy of the informed consent comprehension as a whole. The consent procedure would have been better if the informed consent document is given to the patient to take home, read, and discuss with family members before appending his or her signature. A recent Nigerian study showed that recall of risks was higher in patients who went home with written materials when compared to those who only had verbal counseling [20]. Ezeome et al. in their study on the content of informed consent forms in surgical departments of some Nigerian hospitals found inadequate information on the risks associated with surgery and anesthesia [21]. The use of supplementary materials, especially multimedia, to enhance comprehension of patients has been proven in many studies around the world; these innovations, however, have not been introduced in many Nigerian hospitals [22–24].

In conclusion, this work has highlighted some of the deficiencies in the informed consent procedure in otolaryngology surgery in Nigeria. There is need for a more robust informed consent process that includes the initial outpatient consultation and the preoperative and postoperative period. The importance of written and other forms of communication cannot be overemphasized. It is up to physicians to understand and use these alternative information channels where possible so as to achieve a better level of comprehension among patients. It is also important that the information needs of patients be tailored according to their peculiarities and the need for feedback from patients regarding comprehension of the process.

From the foregoing, it is clear that a lot of gaps still need to be filled in the area of informed consent in the hospital setting in developing countries like Nigeria. The authors believe that this work will, to an extent, contribute to closing that gap.
